# A basic Helix-Loop-Helix (SlARANCIO), identified from a *Solanum pennellii* introgression line, affects carotenoid accumulation in tomato fruits

**DOI:** 10.1038/s41598-019-40142-3

**Published:** 2019-03-06

**Authors:** Vincenzo D’Amelia, Assunta Raiola, Domenico Carputo, Edgardo Filippone, Amalia Barone, Maria Manuela Rigano

**Affiliations:** Department of Agricultural Sciences, University of Naples Federico II, Portici, 80055 Italy

## Abstract

Carotenoid accumulation in tomato (*Solanum lycopersicum*) fruits is influenced by environmental *stimuli* and hormonal signals. However, information on the relative regulatory mechanisms are scanty since many molecular players of the carotenoid biosynthetic pathway are still unknown. Here, we reported a basic Helix-Loop-Helix transcription factor, named SlARANCIO (SlAR), whose silencing influences carotenoid accumulation in tomato fruits. The *SlAR* gene was found in the *S*. *pennellii* introgression line (IL) 12-4SL that holds the carotenoid QTL lyc12.1. We observed that the presence of the wild region in a cultivated genetic background led to a decrease in total carotenoid content of IL12-4SL fruits. To get insights into the function of *SlAR*, a quick reverse genetic approach was carried out. Virus-induced gene silencing of *SlAR* in *S*. *lycopersicum* M82 and MicroTom fruits reproduced the same phenotype observed in IL12-4SL, i.e. decreased content of lycopene and total carotenoids. Vice versa, the overexpression of *SlAR* in *Nicotiana benthamiana* leaves increased the content of total carotenoids and chlorophylls. Our results, combined with public transcriptomic data, highly suggest that *SlAR* acts indirectly on the carotenoid pathway and advances current knowledge on the molecular regulators controlling lyc12.1 and, potentially, precursors of carotenoid biosynthesis.

## Introduction

Carotenoids are hydrophobic pigments (generally orange, yellow and red) mainly synthesized via the plastid-localized methylerythritol-4-phosphate (MEP) pathway^1^. They are essential components of the photosynthetic machinery, where they protect from photoxidative damages and act as accessory pigments in light-harvesting complexes^2,3^. Carotenoids are also important for human nutrition, acting as strong antioxidants and vitamin A precursors. A diet rich in carotenoids is correlated with the risk reduction to develop certain types of cancers, coronary heart diseases and several degenerative pathologies^4^. Their fundamental role in plant physiology and the beneficial effects for human health makes the molecular regulation of carotenoids a topic of great interest for the scientific community and carotenoid accumulation an attractive trait for plant breeding efforts. Tomato (*Solanum lycopersicum*) fruits are excellent candidates for carotenoid fortification, given their worldwide consumption as both fresh and processed products^5^. In this crop, significant progresses have been made to elucidate the carotenoid biosynthetic pathway and the relative structural genes^1^. The biosynthesis of lycopene, the most abundant carotenoid conferring the typical deep red-coloration to ripe tomatoes^6^, has been proposed to proceed from geranylgeranyl diphosphate (GGPP) through a pathway catalysed by the enzymes phytoene synthase (PSY), phytoene desaturase (PDS), 15-cis-zeta-carotene isomerase (Z-ISO), ζ-carotene desaturase (ZDS) and prolycopene isomerase (CrtISO)^7,8^. Once biosynthesized, the lycopene molecule can follow two different routes: one leads mainly to β-carotene through lycopene circularization, catalyzed by lycopene cyclase (LCY-B); the other leads mainly to lutein. Generally, the regulation of carotenoids accumulation is quite complex, being controlled by a plethora of internal and external *stimuli*. In tomato fruits, carotenoid accumulation is influenced by the quantity and the quality of light^7,9,10^. Furthermore, different hormones, including ethylene and auxin, are key players in the environment/fruit ripening interaction^1^. A cross talk between different regulatory genes has been proposed to oversee, either directly or indirectly, the elaborate network controlling carotenoid biosynthesis in tomato fruits, also through the regulation of the incoming flux of precursors^1,11^. However, information on these regulatory genes are still insufficient.

An interesting approach to identify regulatory candidate genes is to screen for those mapping within a major quantitative trait locus (QTL) controlling the trait under study. A valid genetic material for such studies in tomato is represented by wild introgression line (IL) populations^12^. The phenotypic differences observed between different ILs are caused by single genomic regions introgressed from the wild donor into the cultivated genetic background. In tomato, a major QTL for increased lycopene content (named lyc12.1) was detected on chromosome 12 of an IL population derived from a cross between *S*. *lycopersicum* and *S*. *pimpinellifolium*^13,14^. In the introgressed region, Kinkade & Foolad^14^ annotated a 15-cis-ζ-carotene isomerase (*Z-ISO*) gene that is involved in the initial steps of *trans*-lycopene biosynthesis. In the same region, the authors found regulatory candidate genes belonging to MYB and bHLH (basic Helix-Loop-Helix) classes. However, cloning and characterization of these genes have not been reported up to now. More recently, Rigano *et al*.^15^ identified the corresponding lyc12.1 QTL in the introgression line IL12-4 of the *S*. *pennellii* IL population constructed by Eshed and Zamir^16^. The authors found that in IL12-4 the presence of wild alleles deriving from the green-fruited *S*. *pennellii* entailed a reduction of about 36% of total carotenoid content. Currently, it is still not known whether genes coding for transcription factors (TFs) and/or other regulatory genes located in this region on chromosome 12 may influence carotenoid biosynthesis in tomato fruits.

This work was designed to investigate the regulatory genes that could influence the activity of the lyc12-1 QTL. Based on genomic and gene expression data obtained, we focused on the bHLH gene (here named *SlARANCIO*, *SlAR*) mapping in this QTL; its wild allele is present in the *S*. *pennellii* introgression subline IL12-4SL previously obtained in our laboratories^17^. *SlAR* belongs to the XII subfamily of bHLHs which, according to the classification of Heim *et al*.^18^, includes proteins related to light and hormones signaling. To get further information on the role of *SlAR* in tomato carotenoid biosynthesis, we performed the phenotypic and molecular characterization of tomato fruits in which *SlAR* expression was modified through virus-induced gene silencing (VIGS) method. Finally, to further verify the ability of *SlAR* to affect carotenogenesis, we carried out heterologous overexpression of the *SlAR* gene in *Nicotiana benthamiana*.

## Results

### The *S*. *pennellii* introgressed region in IL12-4SL negatively affects carotenoid accumulation in tomato ripe fruits

**Figure 1:**
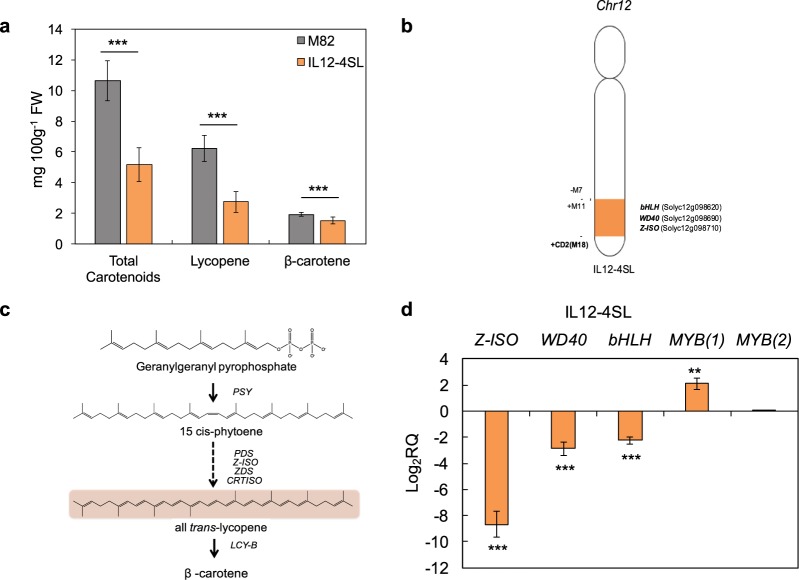
The IL12-4SL holds a *S*. *pennellii* region that negatively influences carotenoid biosynthesis in tomato fruits. (**a**) Total carotenoids, lycopene and β-carotene content (mg 100 g^−1^ FW) in mature red fruits from analyzed tomato lines. (**b**) Schematic representation of the tomato chr12 and position of the wild introgressed region in the subline IL12-4SL (in orange) used in this study. Markers delimiting the borders (+, wild; −, cultivated) and bins are reported. Markers coded “M” are from the work of Ruggieri *et al*.^17^ The position of *Z-ISO* (*Solyc12g098710*) and genes coding for WD40 (*Solyc12g098690*) and bHLH (*Solyc12g098620)*, analysed in this study, is also reported. (**c**) Biosynthetic pathway of carotenoids in tomato. (**d**) Relative mRNA accumulation of the genes coding for *Z-ISO* and for the TFs bHLH, WD40 and the two MYBs in tomato ripe fruits of IL12-4SL compared to M82. In (**a**,**d)**, values are means ± SD (n = 9). Asterisks indicate statistically significant differences in IL12-4SL compared to M82 (***P values < 0.001 according to Student’s *t*-test).

Carotenoid content was monitored in the cultivated parental line M82 and in the introgression subline IL12-4SL (Fig. 1a,b). Compared to M82, IL12-4SL showed a reduction of about 50% of total carotenoids. Consistently, IL12-4SL displayed a reduction of about 50% of the lycopene fraction and a decrease of 25% of β-carotene (Fig. 1a). These results suggest that the tomato genomic region replaced with the corresponding *S*. *pennellii* segment in IL12-4SL influences carotenoid accumulation in mature red fruits (Fig. 1b).

### Candidate genes coding for transcription factors related to carotenoid biosynthesis were identified in IL12-4SL

The *S*. *pennellii* region introgressed in IL12-4SL covers a DNA fraction of 1.8 Mbp^17^ (Fig. 1b). We explored the 180 wild alleles of this region to identify genes potentially involved in the carotenoid biosynthetic pathway. We found the gene *Z-ISO* (*Solyc12g098710*) that encodes the enzyme 15-cis-ζ-carotene isomerase (EC5.2.1.12), an enzyme involved in the upstream steps of the carotenoid biosynthetic pathway before lycopene biosynthesis (Fig. 1c). In close proximity to *Z-ISO*, we identified four putative genes encoding for TFs (Fig. 1b). These TFs were annotated as a bHLH (*Solyc12g098620*), a WD40 (*Solyc12g098690*) and two MYBs (MYB(1), *Solyc12g099140* and MYB(2), *Solyc12g098370*). The relative mRNA levels of these genes were analyzed in mature red fruits of the cultivated M82 and of IL12-4SL. The wild allele of *Z-ISO* was down regulated 8-fold in IL12-4SL compared to M82 (Fig. 1d). Similarly, the genes coding for the transcription factors WD40 and bHLH showed a reduction of the mRNA levels in IL12-4SL *vs* M82 (3-fold and 2-fold, respectively). These results were in line with those previously obtained in the parental line IL12-4^19^. The expression of the genes coding for the two MYBs showed an opposite trend compared to the structural gene *Z-ISO* (Fig. 1d). To understand whether the mRNA expression differences detected for the TF genes were also associated to structural variants, we compared the coding sequences of wild and cultivated alleles (Table 1; Supplementary Table S1). The wild bHLH allele held the highest and variable number of CDS variants. These were one insertion of three triplets (c.148_149insTTGATAATTTTG) and about 19 SNPs (Supplementary Table S1). Among them, particular interesting is the substitution (c.330dupT) that caused a frameshift mutation (Supplementary Table S1). In regard to the *S*. *pennellii* genes coding for the TFs MYBs and WD40, we detected 15 and 6 variants, respectively, with only low or moderate effects (Table 1). Finally, 11 variants were found in the *S*. *pennellii Z-ISO* allele, which were predicted to have low or moderate effects on protein function compared to the cultivated allele.

**Table 1:** CDS structural variants of *S*. *pennellii* alleles identified in the introgressed region IL12-4SL and their effect on protein activity.

Gene name	Solyc ID	Total CDS variants (No.)	Variants with high effect (No.)	Variants with moderate effect (No.)	Variants with low effect (No.)
***MYB***	Solyc12g098370	6	0	3	3
*bHLH*	Solyc12g098620	20	1	8	11
***WD40***	Solyc12g098690	15	0	2	13
*Z-ISO*	Solyc12g098710	11	0	7	4
***MYB***	Solyc12g099140	15	0	6	9

In summary, the wild alleles of *bHLH* and *WD40* showed a gene expression profile consistent with the IL12-4SL phenotype. Further, strong differences were predicted between cultivated and wild alleles of the *bHLH* gene, with consequent impact on the function of the corresponding protein.

### *SlAR* is a tomato TF grouping with the XII bHLH subfamily

**Figure 2:**
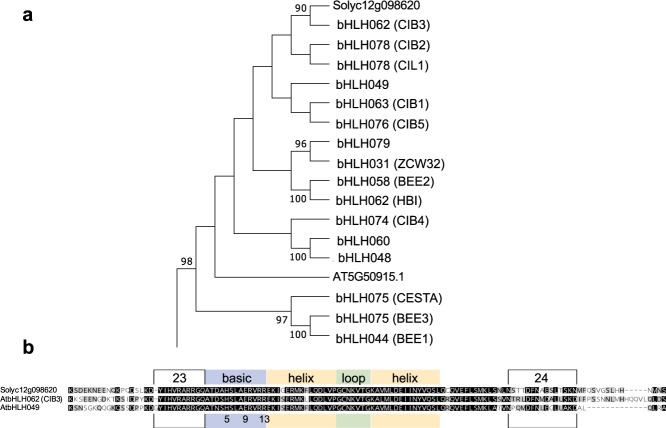
(**a**) Branch of the phylogenetic tree (Supplementary Fig. S1) showing clustering of the tomato bHLH protein (Solyc12g098620) with Arabidopsis bHLH proteins. The bHLH nomenclature is reported and synonym names were assigned to proteins whose function has been previously studied and annotated on TAIR (www.arabidopsis.org). The percent of reliability is labeled next to each branch (bootstraps). Value lower that 70% of bootstrap are not shown. (**b**) Conserved domains of XII family: bHLH motif are displayed by colored box; white boxes named 23-24 indicate bHLH flanking regions typical of the XII subfamily. H(5)E(9)R(13) residues involved in DNA binding are also displayed.

We performed phylogenetic analyses of the WD40 and bHLH predicted proteins to get insights into their possible function in plant. We proceeded with a blast research on Arabidopsis proteome database, because an in-depth analysis of this family has been carried out only in this model plant^18,20–23^. The WD40 protein clustered with the Arabidopsis WD40-transducin family, which is still an uncharacterised group of regulatory TFs (data not shown). More compelling results were obtained for the bHLH that, according to the classification described in Heim *et al*.^18^, clustered with the Arabidopsis subfamily XII (Fig. 2a; Supplementary Fig. S1). In details, the tomato bHLH grouped in the same clade of three cryptochrome interacting bHLHs (CIBs) and, in particular, with CIB3. Consistently with the known characteristics of the XII subfamily, the tomato bHLH showed the typical H(5)-E(9)-R(13) residues for DNA binding in the G-box motif, as well as the presence of conserved amino acid sequences flanking the bHLH domain (named motif^23^ and motif^24^ according to the data of Pires & Dolan; Fig. 2b). Inspecting public transcriptomics datasets, we found that the expression of this bHLH gene in tomato was high in roots and in ripened fruits (Breaker + 10), whereas it was very low in leaves (tomato eFP browser at bar.utoronto.ca). In fruits, the expression of the bHLH gene was lower in the pericarp and higher in locules, placenta and seeds and, generally, it increased with ripening (Supplementary Table S2 and Fig. S2). In locules, placenta and seeds, bHLH was consistently co-expressed (correlation > 0.7) with other genes showing a regulatory function in developmental processes, hormone signalling and stress responses (e.g. NAC, Auxin Responsive SAUR proteins and MADS box; Supplementary Fig. S3). In addition, transcriptomic data showed that the bHLH gene has a correlation of 0.66 with the Geranyl Geranyl pirophospahte synthase gene (*Solyc07g064660*; Supplementary Table S2), codifying for the enzyme involved in the biosynthesis of diterpens (e.g. carotenoids, gibberellin and chlorophylls). Higher correlations (between 0.7 and 0.83) with structural genes not directly related with the carotenoid biosynthetic pathway were found (Supplementary Table S2 and Fig. S4). The majority of these genes are involved in primary metabolism, e.g fatty acid (*Solyc01g008780*, *Solyc06g059710*) and amino acid (*Solyc04g063350* for valine/isoleucine, *Solyc03g112040* for tryptophan) metabolism; hormone biosynthesis, e.g. brassinosteroid (*Solyc05g011970*) and abscisic acid (*Solyc08g075320*); and fruit ripening, e.g. cell wall modification (*Solyc09g010210*, *Solyc06g060170*, *Solyc06g051800*, *Solyc12g010200*). Consistently with the features above observed (i.e. relation with signalling and ripening processes), the cis- elements found in the bHLH promoter region included six light responsive elements, one ethylene responsive element and also one MYB binding site (Table 2).

**Table 2:** List of regulatory elements present within 1,500 bp of *bHLH* (*Solyc12g098620*) promoter.

Motif	Position^a^/(Strand)	Sequence	Function
BOX 1	−247 (−)	TTTCAAA	Light response
BOX 4	−1434 (+)	ATTAAT	Light response
GA-motif	−123 (−)	AAAGATGA	Light response
GATA-motif	−170 (−)	AAGGATAAGG	Light response
GT1-motif	−1489 (−)	GGTTAAT	Light response
Sp1	−1023 (+)	GGGCGG	Light response
MBS	−510 (−)	TAACTG	MYB Binding Site
Skn-1	−488 (−)	GTCAT	Endosperm expression
As-2-box	−808 (−)	GATAatGATG	Shoot-specific expression and light response
W-Box	−776 (+)	TTGACC	WRKY TF Binding Sites
ERE	−825 (−)	ATTTTAAA	Ethylene Responsive Element
CGTCA-motif	−853 (+)	CGTCA	MeJA-responsiveness element
WUN-motif	−821 (+)	AAATTCTT	Wound-responsive element
As1	−853 (−)	TGACG	Oxidative stress-responsive
STRE	−1026 (+)	AGGGG	Stress-responsive element
TC-rich repeats	−805 (+)	ATTCTCTAAC	Defense and stress responsiveness
ARE	−1136 (−)	AAACCA	Anaerobic-induction Element

^a^Number of nucleotides from annotated start codon.

In this paper we renamed the transcription factor bHLH coded by the gene *Solyc12g098620* as *SlARANCIO* (*SlAR*).

### *SlAR* promotes carotenoid biosynthesis in tomato fruits and in a heterologous system

**Figure 3:**
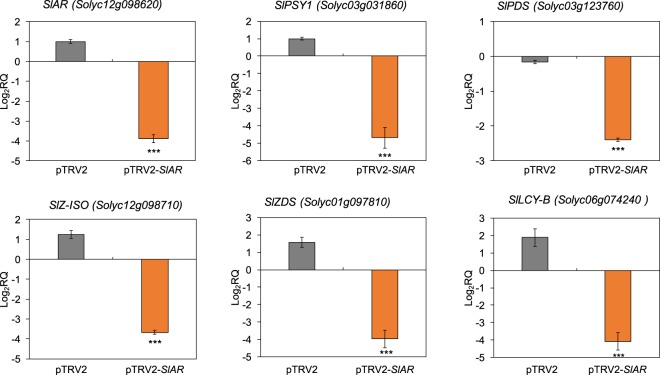
The carotenoid structural genes were down regulated in infiltrated MicroTom fruits where *SlAR* was silenced (VIGS). Relative mRNA levels of *SlAR* and carotenoid structural genes *SlPSY1* (*Solyc03g031860*), *SlPDS* (*Solyc03g123760*), *SlZ-ISO* (*Solyc12g098710*), *SlZDS* (*Solyc01g098710*) and *SlLCY-B* (*Solyc06g074240*) monitored in fruit infiltrated with the empty vector (pTRV2) and with pTRV2-*SlAR* and harvested 10 days after infection. Values are means ± SD (n = 3). Asterisks indicate statistically significant differences in fruits infected with pTRV2-*SlAR* and pTRV2 compared with control (non-infiltrated fruits) (***P value < 0.001 according to Student’s *t*-test).

To quickly verify whether *SlAR* may have some effects on carotenogenesis, we proceeded to its direct functional characterization in tomato fruits. Micro Tom tomatoes infiltrated with the vector pTRV2-*SlAR* exhibited a 4-fold reduction of *SlAR* mRNA levels compared with the control (non-infiltrated samples; Fig. 3). This indicated an efficient silencing of our target. With the reduction of *SlAR* mRNA level, we found a general and significant reduction (P < 0.001) of the expression levels of the major structural genes of the carotenoid biosynthetic pathway and in particular of those leading to lycopene and β-carotene biosynthesis (Fig. 3). The highest down regulation (about 4.7-fold) was found for the fruit specific isoform of phytoene synthase (*SlPSY1 Solyc03g031860*). The enzyme PSY (EC 2.5.1.32) catalyzes the condensation of two molecules of geranylgeranyl diphosphate molecules (GGPPs) and it is considered the major bottleneck in carbon flux to carotenoids^1,24^. Interestingly, we also found that *SlAR* silencing led to the reduction of *WD40* mRNA levels (about 3.5-fold; Supplementary Fig. S5).

**Figure 4:**
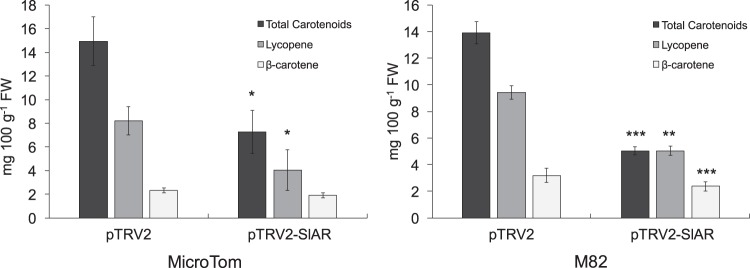
Carotenoids content in *SlAR* silenced tomato fruits. Total carotenoids, lycopene and β-carotene content (mg 100 g^−1^ FW) in MicroTom (**a**) and M82 (**b**) fruits infiltrated with the empty vector (pTRV2) and with pTRV2-*SlAR* and harvested 20 days after infection. Values are means ± SD (n = 3). Asterisks indicate statistically significant differences of total carotenoids, lycopene and β-carotene in fruits infiltrated with pTRV2-*SlAR* compared with fruits infiltrated with pTRV2 (*P < 0.05, **P < 0.01 and ***P values < 0.001 according to Student’s *t*-test).

**Figure 5:**
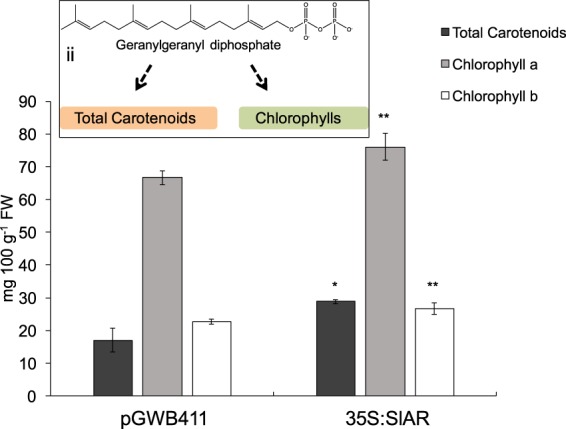
Carotenoid and chlorophyll contents in tissues of *N*. *benthamiana* plant overexpressing *SlAR*. Total carotenoids, chlorophyll a and b in tissues agro-infiltrated with the vector 35S:SlAR and the control (pGWB411 empty vector). Values are means ± SD (n = 3). Asterisks indicate statistically significant differences in leaves infiltrated with pGWB411 empty vector compared with leaves infiltrated with 35S:*SlAR* (*P < 0.05 and **P values < 0.01 according to Student’s *t*-test. ii) the intermediate geranylgeranyl diphosphate for the production of the lipophilic chain (phytol) of chlorophylls is shown.

We next evaluated the metabolic content of MicroTom fruits at the mature red stage. In pTRV2-*SlAR* infiltrated samples, a significant reduction (P < 0.05) of total carotenoids and lycopene contents compared to the pTRV2 infiltrated tomatoes was found (Fig. 4a). A higher significant reduction of total carotenoids (P < 0.001), as well as of the content of the single fractions of lycopene (P < 0.05) and β-carotene (P < 0.001), was evidenced when M82 fruits were used (Fig. 4b). Again, lycopene was the carotenoid with the strongest decline (50% reduction compared to samples infiltrated with the empty vector pTRV2). Interestingly, silencing of *SlAR* induced a very similar visual phenotype in MicroTom and M82 fruits. In particular, a pale red/yellow color was observed in the silenced areas of both lines (Supplementary Fig. S6a,b). To check if *SlAR* can positively affect carotenoid metabolism also in a heterologous system, we performed a transient overexpression in leaves of *N*. *benthamiana* (Fig. 5). Four days after agro-infiltration, we detected a higher content of total carotenoids in tissues agro-infiltrated with the vector carrying 35S:*SlAR* compared to tissues agro-infiltrated with the empty vector (28.8 mg 100 g^−1^ FW *vs* 15 mg 100 g^−1^ FW). We also found an increase of both chlorophyll a (+10%) and chlorophyll b (+4%) content. This further suggested that *SlAR* causes a metabolic boost of the first biochemical steps of carotenogenesis. Indeed, the chlorophyll pathway shares the intermediate geranylgeranyl diphosphate with carotenoids to produce the lipophilic group (phytol) (Fig. 5).

## Discussion

Carotenoid biosynthesis in plants is complex and tightly regulated. A fine cross‐talk between MEP pathways and environmental and developmental controls operates to allow carotenoid accumulation^25^. In tomato fruits, the carotenoid network control seems to be particularly intricate. Carotenoid accumulation occurs during fruit ripening and it is deeply affected by different hormones, including ethylene, auxin, ABA and brassinosteroids^1,26,27^. Molecules that work as mediators between light signals and fruit ripening are also involved in fruit carotenogenesis^9,28^. However, in tomato many components of such network are still unknown. To select TF genes with a putative effect on carotenogenesis, we analysed the expression of the genes that map in the introgressed region of the *S*. *pennellii* subline IL12-4SL. IL12-4SL was previously obtained in our laboratory using the introgression line IL12-4, carrying a QTL (lyc12.1) controlling lycopene accumulation^14,19^. We found that, compared to the parental cultivated line M82, IL12-4SL shows the phenotype already observed in IL12-4 by Rigano *et al*.^19^ i.e. a reduced lycopene content. To explain this finding, we first hypothesized that the structural gene *SlZ-ISO*, mapped in this introgressed region, may hold a wild null allele. However, since results obtained here suggested that *SlZ-ISO* codifies for a potentially functional enzyme and considering that it was down regulated in IL12-4SL, we hypothesize that its transcriptional regulation could have been affected by the *S*. *pennellii* genomic introgression. The dominant mode of inheritance of lyc12.1^14^ supports the hypothesis that a TF may underline the effects of this QTL on carotenoids accumulation. Indeed, it has been evidenced that *cis*-acting loci (such as those controlled by structural genes, e.g. *Z-ISO*) rarely show dominant effects, whereas *trans-*acting loci (i.e. TF) usually do^29^. Based on our genomic and gene expression data, we focused on a bHLH TF coded by the gene *Solyc12g098620*. Its presence was already observed by Kinkade & Foolad^14^ in the lyc12.1 QTL. This gene was previously named *SlbHLH072*^30^. To avoid ambiguity with the *AtbHLH072*, with which the tomato bHLH gene *Solyc12g098620* does not share orthology relationships^30^ (this work), and considering the phenotype we observed in fruits in which this gene was silenced, we decided to rename the bHLH identified as *SlARANCIO* (italian name for *Orange*).

To get insights into *SlAR* function we used VIGS technology, that is considered an efficient and quick method for gene identification and study in tomato and other species^31,32^. Silencing of *SlAR* in fruits of the model genotype MicroTom produced a decrease in mRNA levels of several carotenoid structural genes and, consequently, a total carotenoids reduction in ripe fruits. This result was further confirmed when we used M82 (the cultivated genetic background of IL12-4SL) fruits to silence *SlAR*. After silencing in M82 fruits, we obtained a phenotype similar to that observed in IL12-4SL, i.e. a decrease in lycopene content as well as a pale red colour in ripe tomatoes. This last trait has already been described in IL12-4 fruits^16,33^. We assumed that *SlAR* affects carotenoid biosynthesis with particular effect on lycopene accumulation and hypothesized that the wild *AR12-4* allele contributes to negatively influence the carotenoid biosynthesis in IL12-4 as well as in IL12-4SL fruits. How does *SlAR* influence lycopene biosynthesis in tomato? A first answer to this question is provided by the functional classification of the family this gene belongs to. Indeed, the bHLH proteins are a large family of TFs that control metabolic, physiological and developmental processes in all eukaryotic organisms. They may influence either directly or indirectly carotenoid accumulation. For example, it has been recently evidenced that bHLHs are mediators between light and hormonal signals and carotenoid and tocopherol metabolisms^34^. In the dark and in shading conditions, the phytochrome bHLH interacting factor (PIF1) represses carotenoid biosynthesis mainly by binding to a G–box motif in the promoter of the gene *AtPSY*^9^. In tomato, the heterologous overexpression of the citrus bHLH1, (homologous to an *Arabidopsis* regulator of brassinosteroid) reduced the lycopene content of fruits^35^. More recently, Zhu *et al*.^36^ found another tomato bHLH, *PRE2*-like, affecting fruit chlorophyll and carotenoid accumulation, probably through the interaction with a light signaling network. Consistently with these observations, we found that *SlAR* is phylogenetically related to a group of proteins with a relevant function in light and hormone/light signalling^18,23^. In particular, *SlAR* grouped with the cryptochrome CRY-interacting bHLH (CIB) and CIB-like (CIL) proteins that in tomato, to our knowledge, have not been characterized yet. From studies in Arabidopsis, we mainly know that CIBs are activated by blue-light and interact with CRYs (in particular with CRY2) to transduce light signal in physiological responses such as floral initiation; however, their effect on carotenoid accumulation is still unclear^37–39^. It also should be pointed out that the function of CRY2-mediated system between Arabidopsis and tomato is slightly different. Giliberto *et al*.^40^ observed that a *SlCRY2*-like gene, when overexpressed in tomato, not only had a photomorphogenetic effect (as observed in Arabidopsis), but also led to an overproduction of both lycopene in ripe fruit and chlorophylls in leaves. This is in line with what we observed in overexpression analyses of *SlAR* in *N*. *benthamiana* leaves. Altogether, the evidences we gathered in this work suggest that *SlAR* affects the availability of precursors required for carotenoid biosynthesis, rather than acting directly in the regulation of the carotenoid biochemical pathway. This is also clearly reflected by public transcriptomic data, wherein we did not observe any correlation between *SlAR* and carotenoid structural genes but rather with genes related to primary (amino acid and fatty acid), hormone (gibberellin and auxin) metabolism and ripening processes (cell wall modification and ethylene response). These aspects are in line with the role of the component of the bHLH subfamily XII, the group our *SlAR* belongs to. Indeed, it has been previously observed that these bHLHs (through CRY-mediated pathways) may influence not only carotenoid accumulation but also have effects on hormones, other antioxidant metabolites and developmental processes^41^.

In conclusion, the exploitation of the genetic resources available today for tomato (i.e. the *S*. *pennellii* introgression lines) combined with fast and efficient techniques for transient transformation allowed the rapid identification of a novel candidate gene influencing the accumulation of carotenoids. We believe three are the most important outcomes of our research: (1) silencing of a bHLH transcription factor, identified in the introgression line IL12-4SL and named *SlAR*, negatively influences carotenoid (in particular lycopene) biosynthesis in tomato fruits, whereas its overexpression induces carotenoid and chlorophyll accumulation in heterologous system; (2) the presence of the wild *S*. *pennellii* allele of *SlAR* may contribute, together with other genes, to the phenotype observed in the introgression line IL12-4SL; (3) *SlAR* may indirectly influence carotenogenesis acting upstream the carotenoid pathway; consequently, it may also affect chlorophyll accumulation. Future physiological studies aimed at understanding how *SlAR* may influence the biosynthesis of carotenoids and their precursors in response to external and internal stimuli are now needed. Subsequent investigations on the involvement of *SlAR* in tomato ripening processes are equally important. They will allow getting additional insights into the multi-level connections existing between these different biochemical and developmental processes.

## Materials and Methods

### Plant Materials

The cultivated genotype M82 (accession LA3475) was kindly provided by the Tomato Genetics Resources Center (http://tgrc.ucdavis.edu/index.cfm), whereas the subline named IL12-4SL was obtained as described in Ruggieri *et al*.^17^. The size of the wild genomic region in the line IL12-4SL, the description of the molecular markers used to define the introgressed region and the annotations and positions of the genes mapping in the wild introgressed region are described in the papers Ruggieri *et al*.^17^ and Rigano *et al*.^19,42^. Plants of each genotype were grown in Acerra (Naples, Italy), in a randomized complete block design with three replications (10 plants/replication). Fruits were collected at Mature Red (55 days post anthesis) stage. Seeds and columella were subsequently removed, and fruits were chopped, ground in liquid nitrogen by a blender (FRI150, Fimar) to a fine powder and kept at −80 °C until the subsequent analyses. For VIGS (Virus-induced gene silencing) the cultivars MicroTom and M82 were used. These plants were grown in a greenhouse at the Department of Agricultural Sciences of the University of Naples (Naples, Italy) in controlled conditions and under natural photoperiod. *Nicotiana benthamiana* plants were used for transient expression analysis and kept in controlled temperature condition (25 °C) under 16/8 h (light/dark) photoperiod. Plants were grown in 20 cm pots containing a 1:1 mixture of medium-sandy soil and compost with three replicates per genotype.

### RNA extraction and gene expression analyses (RT-qPCR)

Total RNA was isolated from plant materials by using the TRIzol^®^ reagent (Invitrogen, Carlsbad, CA, USA) and treated with RNase-free DNase (Invitrogen, Carlsbad, CA, USA; Madison, WI, USA) according to the method reported by the manufacturer (Invitrogen). Total RNA (1 μg) was retrotranscribed by the Transcriptor High Fidelity cDNA Synthesis Kit (Roche) and cDNA was stored at −20 °C until RT-qPCR analysis. For each RT-qPCR reaction, 1 μl of cDNA diluted 1:10 was mixed with 12.5 μl SYBR Green PCR master mix (Applied) and 5 pmol each of forward and reverse primers in a final volume of 25 μl. The reaction was carried out by using the 7900HT Fast-Real Time PCR System (Applied Biosystems). The amplification program was carried out according to the following steps: 2 min at 50 °C, 10 min at 95 °C, 0.15 min at 95 °C and 1 min at 60 °C for 40 cycles. All reactions were run in triplicate for each of the three biological replicates and a reference gene coding for the *elongation factor 1-alpha* (*Ef 1- α* – *Solyc06g005060*) was used as reference gene for tomato samples^43^. A melting curve analysis of the PCR products was produced to verify primer specificity. Primer pairs used for RT-qPCR analyses are listed in Supplementary Table S3. Results were then analysed using the ABI PRISM 7900HT Sequence Detection System Version 2.1 (www.thermofisher. com). The relative expression was estimated according to the ΔΔCt method^44^.

### Virus-induced gene silencing (VIGS)

Fragments of the target gene *Solyc12g098620* (300 bp) of the complementary DNA was amplified with **attB** primers (Forward: 5′- **GGGGACAAGTTTGTACAAAAAAGCAGGCTTC**TTATCTAATCTGAATTCAACAA-3′; Reverse: 5′-**GGGGACCACTTTGTACAAGAAAGCTGGGTC**TTGGCTAAACCCCATATGGAT-3′) and recombined into the vector pDONR207 by the BP reaction to generate an entry clone. The nucleotide sequence of the fragment used for silencing showed no sequence similarity with non-target homologus genes. This was verified through alignment with tomato database (available at: http://solgenomics.net/) and online VIGS tool (available at: http://vigs.solgenomics.net/).

The entry vector was then recombined with the pTRV2-GW destination vector^45^ using an LR reaction to get the VIGS clone pTRV2-*SlAR*. The sequenced VIGS vectors were then transferred into *Agrobacterium tumefaciens* strain GV3101:pMP90 by electroporation. Both control (pTRV2 empty vector) and pTRV2-*SlAR* were co-infiltrated with pTRV1 as described by Orzaez *et al*.^45^. Fruits were infiltrated and marked at the mature green stage, and samples were collected 10 days after infection for transcriptomic studies and 20 days after infection for metabolic analyses. For all analyses, three biological replicates (each consisting of a pool of three fruits) were used. Fruits infected with pTRV2, pTRV2-*SlAR* and non-infiltrated fruits (control) were collected, ground in liquid nitrogen and stored at −80 °C until subsequent analyses.

### Ectopic expression of tomato transcription factors in *N*. *benthamiana* plants

The *SlAR* coding sequence (CDS) was cloned in the 35 SCaMV expression cassette of pGWB411^46^ using Gateway recombination technology (Invitrogen, www.invitrogen.com). **AttB** primers used to clone the entire CDS were: Forward 5′-**GGGGACAAGTTTGTACAAAAAAGCAGGCTTC**ATGGAGAAGAAAAATTTGTTCTT-3′ and Reverse 5′-**GGGGACCACTTTGTACAAGAAAGCTGGGTC**TCATAGTTCAGCCTTCATTTGT-3′. *A*. *tumefaciens* strain GV3101:pMP90, transformed with the expression vectors (pGWB411 and 35 S:*SlAR*), was used for agroinfiltration in fully expanded leaves of *N*. *benthamiana* as reported by D’Amelia *et al*.^47^. As reported by Leonelli *et al*.^48^, *N*. *benthamiana* is an efficient system to study candidate genes of the carotenoid biosynthetic pathway, also when heterologous genes are investigated. Leaves agro-infiltrated with pGWB411, 35S:*SlAR412-4* and non-agroinfiltrated leaves were collected, ground in liquid nitrogen and stored at −80 °C until subsequent analyses.

### Carotenoid and Chlorophyll Determination

The evaluation of total carotenoids, lycopene and β-carotene was carried out according to the method reported by Wellburn^49^ and by Zouari *et al*.^50^ with slight modifications reported by Rigano *et al*.^15^. Briefly, one gram of frozen powder was extracted with a solution of acetone/hexane (40/60, v/v) for 15 min. The mixture was centrifuged at 4000 rpm for 10 min and the absorbance of surnatant was measured at 663, 645, 505, and 453 nm. Chlorophylls a and b content was evaluated according to Zouari *et al*.^50^. Results were expressed as milligrams per 100 g fresh weight (FW). All biological replicates were analyzed in triplicate.

### Bioinformatic and statistical analysis

Arabidopsis bHLH protein sequences were retrieved from plant TF database (http://planttfdb.cbi.pku.edu.cn). Alignment with MUSCLE was performed. MEGA7^51^ was first used to establish the best-fit model of evolution through the option “Find best DNA/Protein Models” implemented in the program. Finally, a Neighbor-joining phylogenetic tree with the appropriate options was constructed with 1,000 bootstrap replicates. Publically available RNAseq data set deposited in Tomato Expression Atlas **(**http://tea.solgenomics.net)^52^ and tomato efp browser (http://bar.utoronto.ca/efp_tomato/cgi-bin/efpWeb.cgi) were used for transcriptional profiling and correlation analysis during fruit ripening of *S*. *lycopersicum* cultivars M82 and Heinz. EXPath 2.0 database (http://expath.itps.ncku.edu.tw/index.html) was used to identify in which metabolic pathway the not annotated structural genes were involved in (Zheng *et al*.^53^). The accession numbers of the proteins used are reported in Supplementary Fig. S1.

Experiments consisting of three or more conditions were tested for statistical significance using one‐way analysis of variance followed by Tukey’s test to compare mean values. Experiments with only two comparisons were tested for significance using Student’s t‐test. Promoter sequence of the transcription factor *SlAR* (*Solyc12g098620*) was obtained using the SolGenomic database https://solgenomics.net. The 1,500 bps upstream the transcription start was analysed using the online software PLANTCARE^54^. The PROVEAN protein tool (http://provean.jcvi.org7seq_submit.php) was used to predict the potential effects of polymorphisms between the wild and the cultivated candidate genes on the protein sequences in terms of neutral or deleterious effects.

## Supplementary information

supplementary information

Supplementary Dataset 2
