# Supplementary material for: A basic Helix-Loop-Helix (SlARANCIO), identified from a Solanum pennellii introgression line, affects carotenoid accumulation in tomato fruits
Source: Sci Rep. 2019 Mar 6;9:3699. doi: 10.1038/s41598-019-40142-3 (PMC6403429; doi:10.1038/s41598-019-40142-3)
Supplement: Supplementary file 1 — supplementary information [file 41598_2019_40142_MOESM1_ESM.pdf]

Supplementary Figures

Supplementary Figures

A basic Helix-Loop-Helix (*SlARANCIO*), identified from a *Solanum pennellii* introgression line, affects carotenoid accumulation in tomato fruit

Vincenzo D'Amelia, Assunta Raiola, Domenico Carputo, Edgardo Filippone, Amalia Barone, Maria Manuela Rigano*

Department of Agricultural Sciences, University of Naples Federico II, Portici, 80055, Italy.
*Contact: Maria Manuela Rigano. Tel: +39 081 2532125. Email: mrigano@unina.it

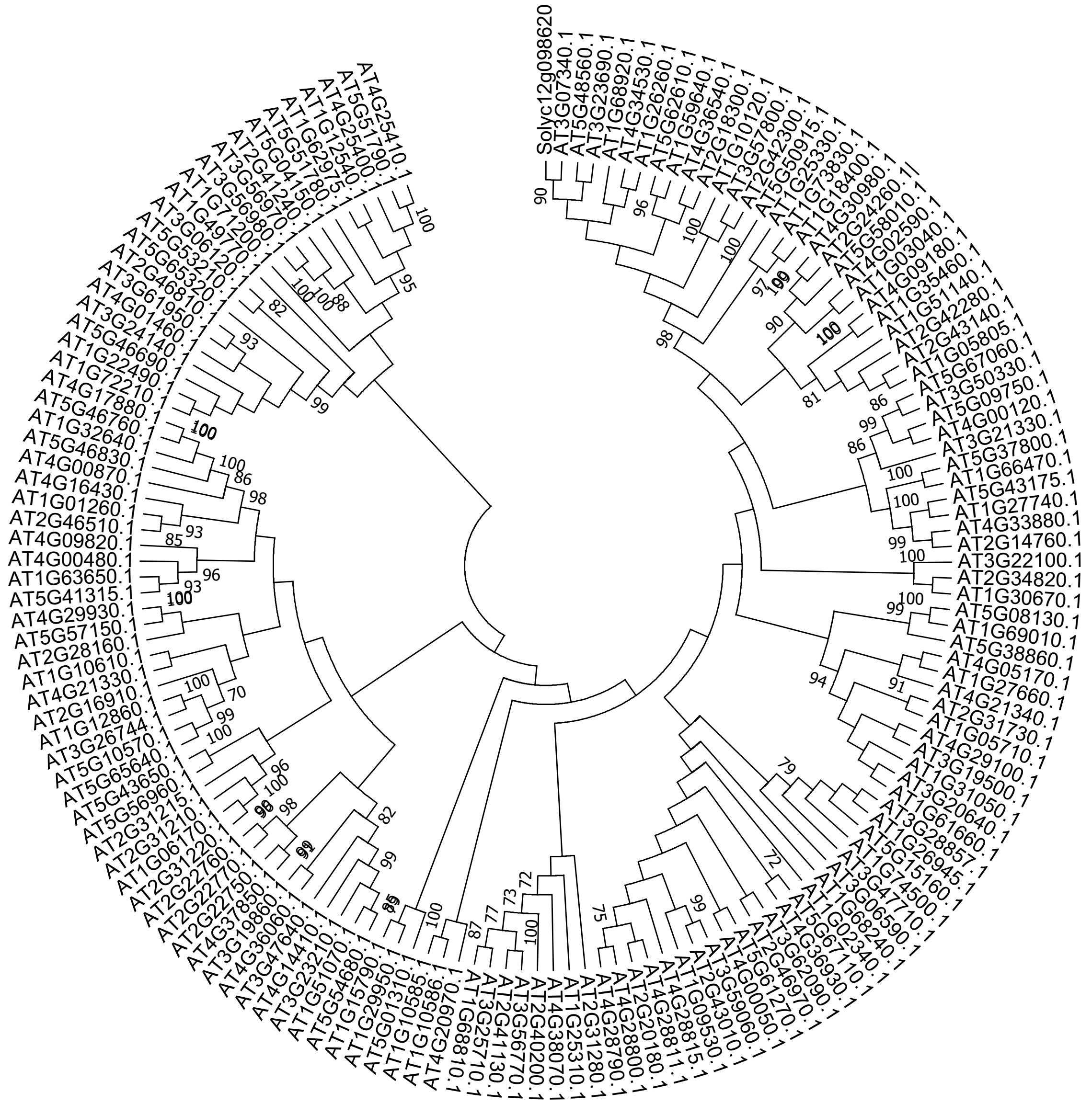

Supplementary Fig. S1. Neighbor-Joining Phylogenetic Tree of the AtbHLHs with the tomato bHLH (Solyc12g098620). The percent of reliability is labeled next to each branch (1,000 replicates). Values lower than 70% of bootstrap are not shown.

Supplementary Figures

A basic Helix-Loop-Helix (*SlARANCIO*), identified from a *Solanum pennellii* introgression line, affects carotenoid accumulation in tomato fruit

Vincenzo D'Amelia, Assunta Raiola, Domenico Carputo, Edgardo Filippone, Amalia Barone, Maria Manuela Rigano*

Department of Agricultural Sciences, University of Naples Federico II, Portici, 80055, Italy.
*Contact: Maria Manuela Rigano. Tel: +39 081 2532125. Email: mrigano@unina.it

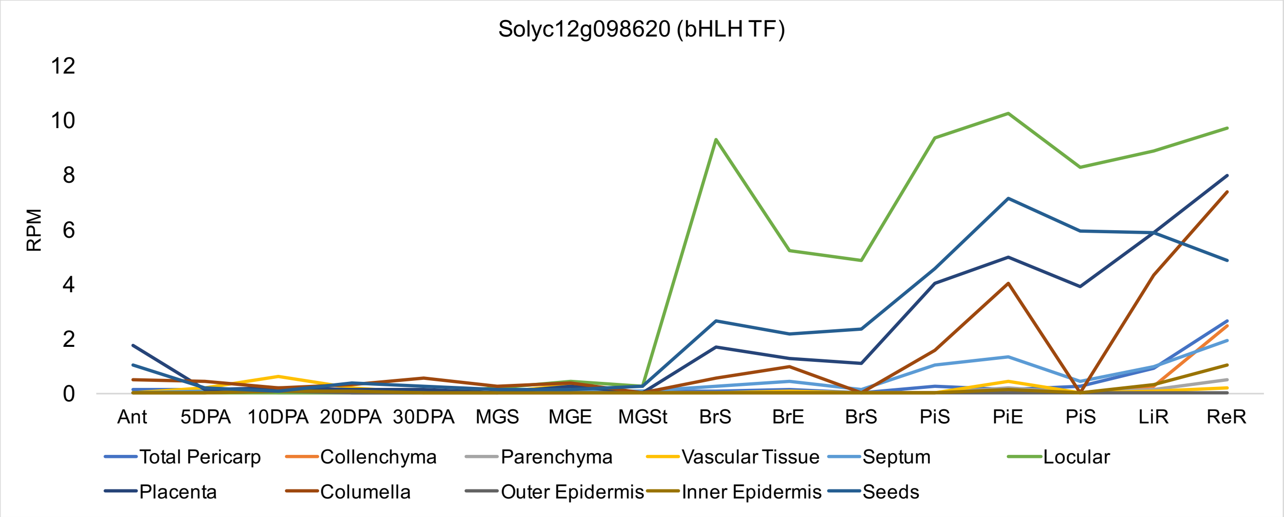

Supplementary Fig. S2. Expression profiles at the mRNA level of the bHLH Solyc12g098620 in all tomato tissues including fruit ripening stages. Expression values were obtained by the public transcriptomic database Tomato Expression Atlas (http://tea.solgenomics.net/) and graphically modified by the authors to obtain a line graph. Ant, Anthesis; DPA, Days Post Anthesis; MGS, Mature Green Stem; MGE, Mature Green Equatorial; MGSt, Mature Green Stylar; BrS, Breaker Stem; BrE, Breaker Equatorial; BrS, Breaker Stylar; PiS, Pink Stem; PiE, Pink Equatorial; PiS, Pink Stylar; LiR, Light Red; ReR, Red Ripe. Tomato Expression Atlas images by TEA are licensed under a Creative Commons Attribution 4.0 International License. Based on work at tea.solgenomics.net.

Supplementary Figures

A basic Helix-Loop-Helix (*SlARANCIO*), identified from a *Solanum pennellii* introgression line, affects carotenoid accumulation in tomato fruit

Vincenzo D'Amelia, Assunta Raiola, Domenico Carputo, Edgardo Filippone, Amalia Barone, Maria Manuela Rigano*

Department of Agricultural Sciences, University of Naples Federico II, Portici, 80055, Italy.
*Contact: Maria Manuela Rigano. Tel: +39 081 2532125. Email: mrigano@unina.it

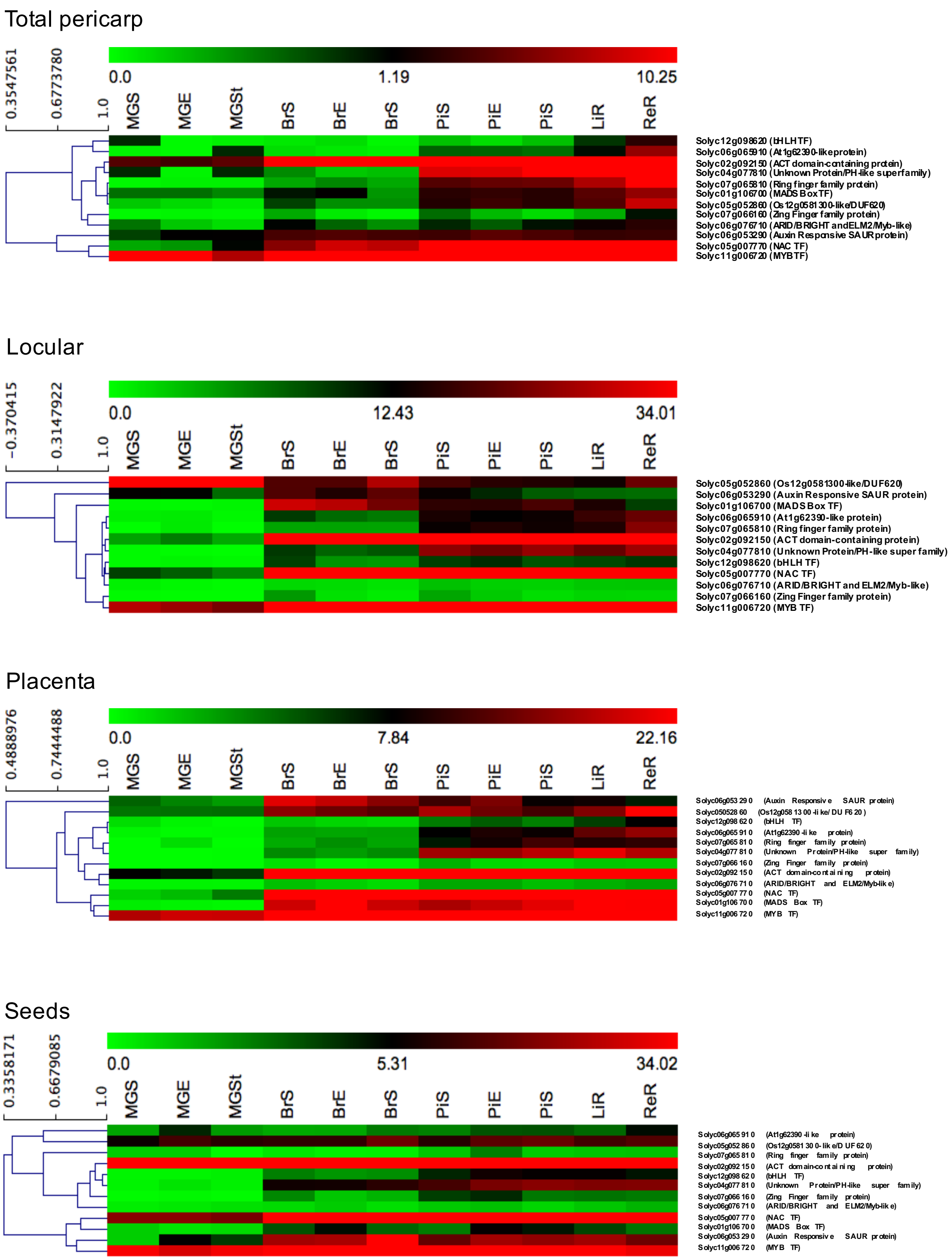

Supplementary Fig. S3. HeatMap showing co-expressed regulatory genes (correlation > 0.7) with bHLH Solyc12g098620 in three fruit tissues and in different fruit ripening stages. The color scale indicates RPM (Reads per million mapped reads) expression values. These values were obtained by the public transcriptomic database Tomato Expression Atlas (http://tea.solgenomics.net/) and graphically modified by the authors to obtain a HeatMap representation. MGS, Mature Green Stem; MGE, Mature Green Equatorial; MGSt, Mature Green Stylar; BrS, Breaker Stem; BrE, Breaker Equatorial; BrS, Breaker Stylar; PiS, Pink Stem; PiE, Pink Equatorial; PiS, Pink Stylar; LiR, Light

Red; ReR, Red Ripe. Tomato Expression Atlas images by TEA are licensed under a Creative Commons Attribution 4.0 International License. Based on work at tea.solgenomics.net.

Supplementary Figures

A basic Helix-Loop-Helix (*SlARANCIO*), identified from a *Solanum pennellii* introgression line, affects carotenoid accumulation in tomato fruit

Vincenzo D'Amelia, Assunta Raiola, Domenico Carputo, Edgardo Filippone, Amalia Barone, Maria Manuela Rigano*

Department of Agricultural Sciences, University of Naples Federico II, Portici, 80055, Italy.
*Contact: Maria Manuela Rigano. Tel: +39 081 2532125. Email: mrigano@unina.it

Total Pericarp

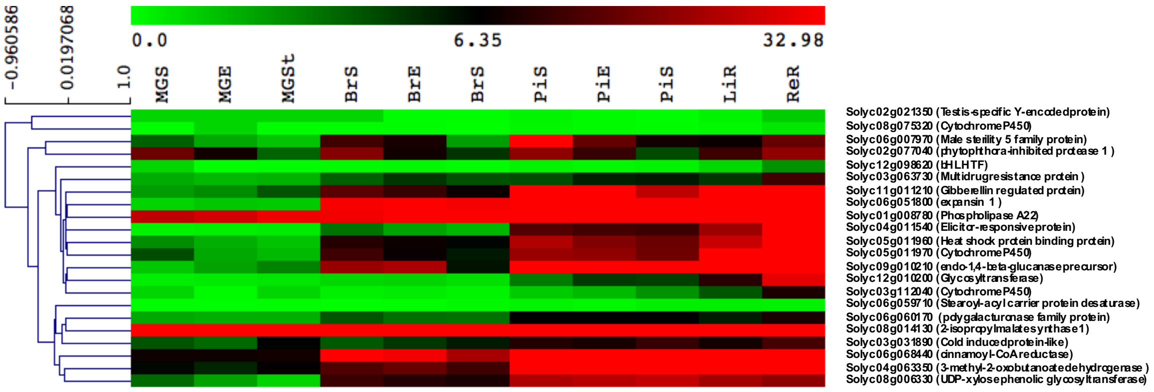

Locular

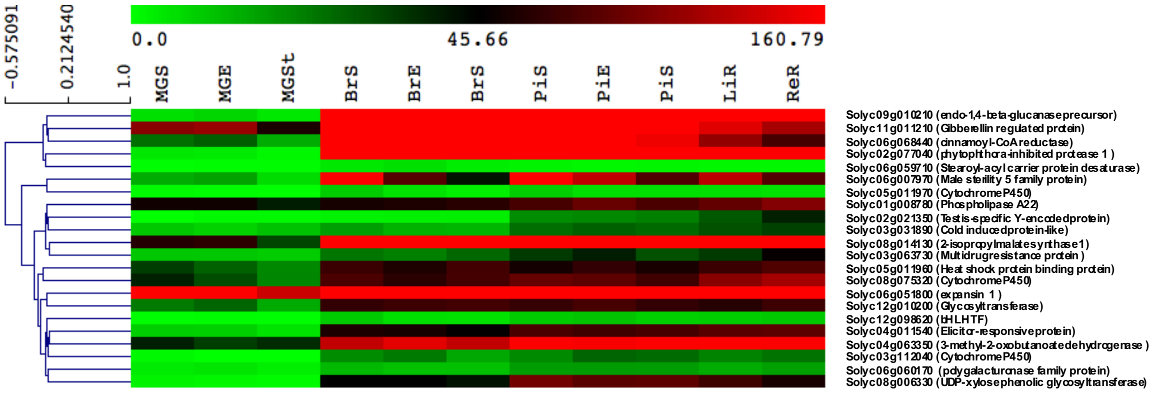

Placenta

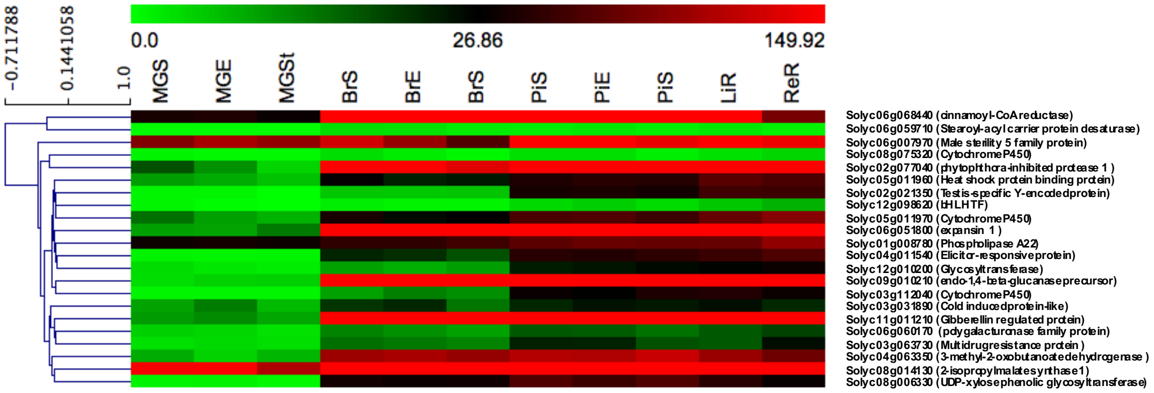

Seeds

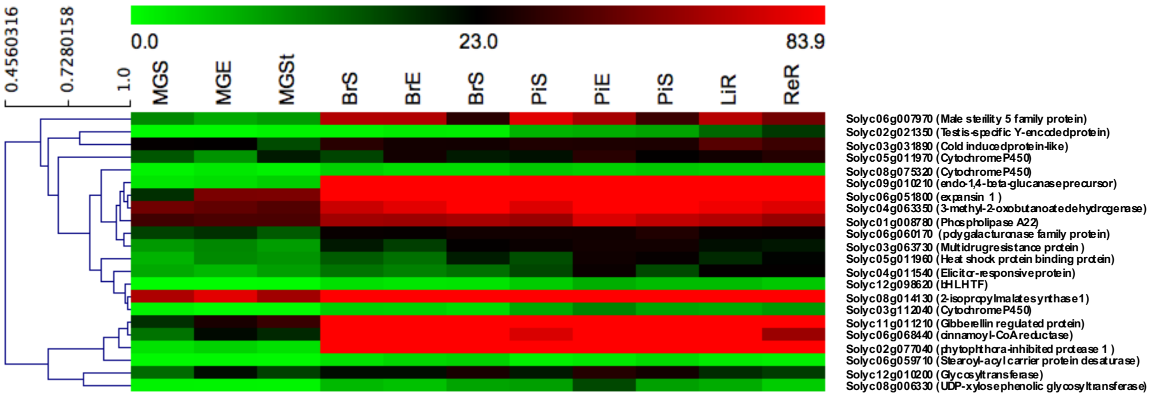

Supplementary Fig. S4. Heat Map showing co-expressed structural genes (correlation > 0.7) with bHLH Solyc12g098620 in three fruit tissues and in different fruit ripening stages. The color scale indicates RPM (Reads per million mapped reads) expression values. These values were obtained by the public transcriptomic database Tomato Expression Atlas (http://tea.solgenomics.net/) and graphically modified by the authors to obtain a HeatMap representation. MGS, Mature Green Stem; MGE, Mature Green Equatorial; MGSt, Mature Green Stylar; BrS, Breaker Stem; BrE, Breaker Equatorial; BrS, Breaker Stylar; PiS, Pink Stem; PiE, Pink Equatorial; PiS, Pink Stylar; LiR, Light Red; ReR, Red Ripe. Tomato Expression Atlas images by TEA are licensed under a Creative Commons Attribution 4.0 International License. Based on work at tea.solgenomics.net.

Supplementary Figures

A basic Helix-Loop-Helix (*SlARANCIO*), identified from a *Solanum pennellii* introgression line, affects carotenoid accumulation in tomato fruit

Vincenzo D'Amelia, Assunta Raiola, Domenico Carputo, Edgardo Filippone, Amalia Barone, Maria Manuela Rigano*

Department of Agricultural Sciences, University of Naples Federico II, Portici, 80055, Italy.
*Contact: Maria Manuela Rigano. Tel: +39 081 2532125. Email: mrigano@unina.it

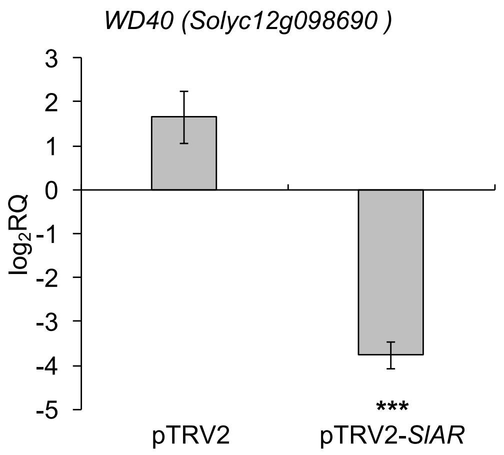

Supplementary Fig. S5. Relative RNA accumulation of the gene *Solyc12g098690* coding for the TF WD40 in control (pTRV2 empty vector) and *SlAR* silenced fruits harvested 10 days after infection compared to M82. Values are means ± SD (n = 9). Asterisks indicate statistically significant differences in fruits infected with pTRV2-*SlAR* and pTRV2 compared with control (non-infiltrated fruits) (****P* values <0.001 according to Student *t*-test).

Supplementary Figures

A basic Helix-Loop-Helix (*SlARANCIO*), identified from a *Solanum pennellii* introgression line, affects carotenoid accumulation in tomato fruit

Vincenzo D'Amelia, Assunta Raiola, Domenico Carputo, Edgardo Filippone, Amalia Barone, Maria Manuela Rigano*

Department of Agricultural Sciences, University of Naples Federico II, Portici, 80055, Italy.
*Contact: Maria Manuela Rigano. Tel: +39 081 2532125. Email: mrigano@unina.it

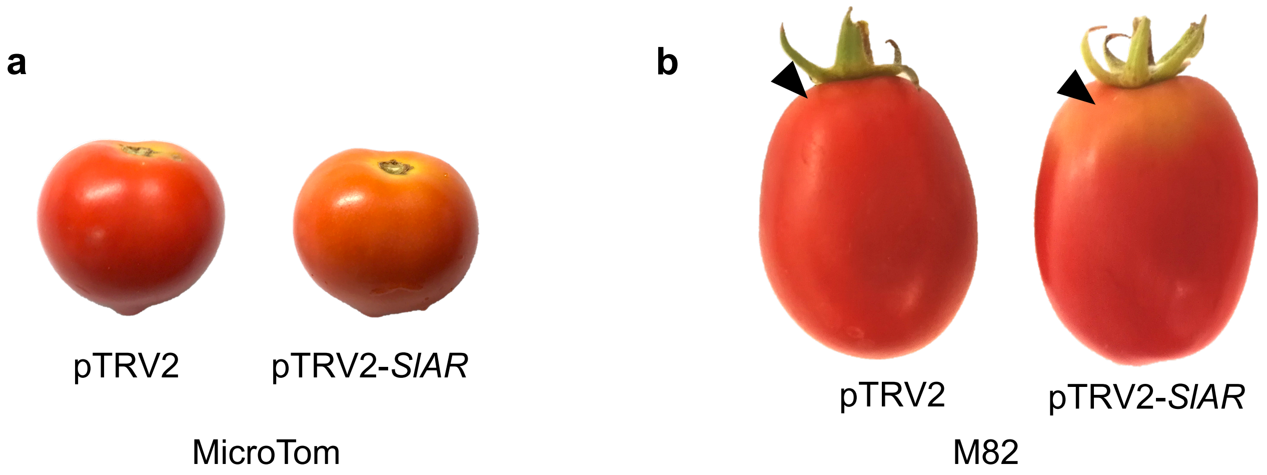

Supplementary Fig. S6. Phenotypes of MicroTom and M82 fruits in which *SlAR12-4* was transiently silenced (VIGS). (A) Phenotype of *SlAR12-4* transiently silenced MicroTom fruits and control (pTRV2) 20 days after infection. (B) Phenotype of *SlAR12-4* silenced M82 fruits and control (pTRV2) 20 days after infection. The silencing effect in M82 was localized especially in upper parts, as shown by black arrows.

Supplementary Material

A basic Helix-Loop-Helix (SlARANCIO), identified from a *Solanum pennellii* introgression line, affects carotenoid accumulation in tomato fruits

Vincenzo D'Amelia, Assunta Raiola, Domenico Carputo, Edgardo Filippone, Amalia Barone, Maria Manuela Rigano*

Department of Agricultural Sciences, University of Naples Federico II, Portici, 80055, Italy.
*Contact: Maria Manuela Rigano. Tel: +39 081 2532125. Email: mrigano@unina.it

Table S1. Selected genes mapping in IL12-4SL and predicted polymorphisms between the wild and the cultivated candidate alleles and the potential effects on the protein sequence.

ID	Effect type	Effect	Nt Change	AA change
Solyc12g098370 (MYB)	disruptive_inframe_deletion	Moderate	c.71_73delATC	p.His24del
	missense_variant	Moderate	c.208T>A	p.Ser70Thr
	synonymous_variant	Low	c.225A>G	p.Pro75Pro
	synonymous_variant	Low	c.234T>A	p.Ala78Ala
	synonymous_variant	Low	c.714C>T	p.Val238Val
	missense_variant	Moderate	c.902G>A	p.Ser301Asn
Solyc12g098620 (bHLH)	synonymous_variant	Low	c.81C>T	p.Pro27Pro
	synonymous_variant	Low	c.117C>T	p.Asp39Asp
	disruptive_inframe_insertion	Moderate	c.148_149insTTGATAATTTTG	p.Phe49_Gly50insValAspAsnPhe
	missense_variant	Moderate	c.268C>T	p.Pro90Ser
	missense_variant	Moderate	c.275C>T	p.Thr92Ile
	frameshift_variant	High	c.330dupT	p.Gly111fs
	synonymous_variant	Low	c.501G>A	p.Gly167Gly
	synonymous_variant	Low	c.582A>C	p.Ser194Ser
	missense_variant	Moderate	c.589G>A	p.Val197Ile
	synonymous_variant	Low	c.663T>A	p.Ala221Ala
	synonymous_variant	Low	c.858A>G	p.Leu286Leu
	synonymous_variant	Low	c.903G>A	p.Thr301Thr
	splice_region_variant &intron_variant	Low	c.913-7T>A	
	missense_variant&splice_region_variant	Moderate	c.915G>C	p.Met305Ile

	missense_variant	Moderate	c.1006T>C	p.Ser336Pro
	synonymous_variant	Low	c.1017A>G	p.Gln339Gln
	splice_region_variant &intron_variant	Low	c.1096-3delT	
	missense_variant	Moderate	c.1117C>A	p.His373Asn
	synonymous_variant	Low	c.1167A>G	p.Gln389Gln
Solyc12g098690 (WD40)	synonymous_variant	Low	c.114C>T	p.Ser38Ser
	synonymous_variant	Low	c.177A>G	p.Thr59Thr
	synonymous_variant	Low	c.186T>C	p.Ser62Ser
	splice_region_variant&intron_variant	Low	c.346-4A>G	
	splice_region_variant&intron_variant	Low	c.480+6C>A	
	synonymous_variant	Low	c.570T>C	p.Phe190Phe
	synonymous_variant	Low	c.633A>T	p.Thr211Thr
	missense_variant	Moderate	c.642A>T	p.Glu214Asp
	missense_variant	Moderate	c.880A>G	p.Ile294Val
	synonymous_variant	Low	c.888A>C	p.Ser296Ser
	synonymous_variant	Low	c.909T>C	p.Tyr303Tyr
	synonymous_variant	Low	c.951C>A	p.Ala317Ala
	synonymous_variant	Low	c.960T>C	p.Asn320Asn
	synonymous_variant	Low	c.1206C>T	p.Tyr402Tyr
	splice_region_variant&stop_retained_variant	Low	c.1290G>A	p.Ter430Ter
Solyc12g098710 (Z-ISO)	missense_variant	Moderate	c.40A>C	p.His13Pro
	missense_variant	Moderate	c.54C>A	p.His18Asn
	missense_variant	Moderate	c.61A>G	p.Lysr20Arg
	missense_variant	Moderate	c.312G>A	p.Val104Leu
	missense_variant	Moderate	c.413A>C	p.Ile151Leu
	synonymous_variant	Low	c.619T>C	p.Ser206Ser
	synonymous_variant	Low	c.905C>T	p.Ala301Ala
	missense_variant	Moderate	c.951A>G	p.Ile317Val
	synonymous_variant	Low	c.968C>T	p.Ala322Ala
	missense_varian	Moderate	c.1004A>G	p.Thr348Ala
	synonymous_variant	Low	c.1053T>C	p.Leu351Leu
Solyc12g099140 (MYB)	missense_variant	Moderate	c.53A>G	p.Asn18Ser
	synonymous_variant	Low	c.138C>T	p.Phe46Phe
	synonymous_variant	Low	c.249T>C	p.Tyr83Tyr
	synonymous_variant	Low	c.276T>C	p.Asn92Asn

synonymous_variant	Low	c.321C>T	p.Arg107Arg
synonymous_variant	Low	c.357G>A	p.Leu119Leu
synonymous_variant	Low	c.381A>T	p.Ile127Ile
synonymous_variant	Low	c.438G>C	p.Val146Val
synonymous_variant	Low	c.462G>A	p.Thr154Thr
missense_variant	Moderate	c.541C>G	p.Leu181Val
missense_variant	Moderate	c.673A>G	p.Ile225Val
missense_variant	Moderate	c.796G>T	p.Asp266Tyr
missense_variant	Moderate	c.869G>T	p.Ser290Ile
disruptive_inframe_insertion	Moderate	c.893_895dupAAC	p.Gln298dup

Supplementary Tables

A basic Helix-Loop-Helix, *SlARANCIO*, identified from a *Solanum pennellii* introgression line, affects carotenoid accumulation in tomato fruits

Vincenzo D'Amelia, Assunta Raiola, Domenico Carputo, Edgardo Filippone, Amalia Barone, Maria Manuela Rigano*

Department of Agricultural Sciences, University of Naples Federico II, Portici, 80055, Italy.

*Contact: Maria Manuela Rigano. Tel: +39 081 2532125. Email: mrigano@unina.it.

Table S3. List of primer used for RT-qPCR analyses.

Gene	Primer
Solyc12g098710* *(Cis-Z-Isomerasi)	FW: 5' - TTCCCTCAAACCCCAGAAAG -3'
	REV: 5' - CCCAGCAAAATAAACCCAAG- 3'
Solyc12g098620* *(bHLH)	FW: 5'- GATTATATTCATGTTAGAGCTAGAAGGG - 3'
	REV: 5'- CATCACAGCTTTTCCAGTCAC -3'
Solyc12g098690* *(WD40)	FW: 5'- CGAATTTGGGACATGAGAAACC -3'
	REV: 5'- GAGTAACGGATTGATCGGACAG-3'
Solyc06g005060* *(Endogenous Factor EF-α)	FW: 5'-CAACCCTGACAAAATCCCCTTT-3'
	REV:5'-TTGGTCCCTTGTACCAGTCGAG-3'
Solyc12g099140* *(Myb)	FW: 5' - GGGCAATAGGTGGTCGAA-3'
	REV: 5' - TTGGACTCGAGTTCTCCAGTAAT-3'
Solyc12g098370* *(Myb)	FW: 5' - GGGTGAAAGGACTCACCCTA-3'
	REV: 5' - TTTATAGCTCTCGGCTGCCT-3'
Solyc03g031860	FW: 5'- GAAGATGTTTTCAATGGGCGG-3'
(PSY)	REV: 5' – TCTCAAGTCCATACGCATTCC- 3'
Solyc03g123760	FW: 5'- TGGGTGGTTTGTCTACAGCAAA-3'
(PDS)	REV: 5'- ATCCCTTGCCTCCAGCAGTA-3'
Solyc01g098710	FW: 5' - TTGGAGCGTTCGAGGCAAT-3'
(ZDS)	REV: 5' - AGAAATCTGCATCTGGCGTATAGA-3'
Solyc06g074240	FW: 5' - TGTTATTGAGGAAGAGAAATGTGTGAT-3'
(CYCB)	REV: 5' - TCCCACCAATAGCCATAACATTTT-3'
